# Exosomes Derived From Senescent Cells Promote Cellular Senescence

**DOI:** 10.1093/geroni/igaa057.435

**Published:** 2020-12-16

**Authors:** Genxiang Mao, Xiaogang Xu

**Affiliations:** 1 Zhejiang Hospital, hangzhou, Zhejiang Province, China; 2 Zhejiang Hospital, Hangzhou, Zhejiang, China

## Abstract

Exosomes are one type of small-cell extracellular vesicles (sEVs), which together with the senescence-associated secretory phenotype (SASP) mainly constitute the senescent microenvironment and perform remotely intercellular communication. However, the effects of senescence on exosomes biosynthesis and secretion and its role in the cell senescence are still obscure. Here, we used human fetal lung diploid fibroblasts (2BS) passaged to PD50 to construct the senescent cells model in vitro, which were confirmed by senescence-related β-galactosidase staining, cell cycle distribution, and intracellular ROS levels. PD30 2BS was used as young control. We evaluated the exosomes derived from senescence and young control group respectively and investigated their regulation of senescence. We found that exosomes released from 2BS had typical sizes and cup-shapes morphology and their surface presented typical exosome-associated proteins. The number of exosomes secreted by senescent cells was significantly higher than that of young cells. Moreover, exosomal markers Alix, TSG101, and CD63 were all more expressed than young cells. Furthermore, we treat young cells with exosomes secreted by senescent cells, which can induce senescence-like changes in young cells, including increased SA-β-Gal activity, up-regulated p16 protein expression, and activation of the Notch signaling pathway. The above results imply that exosomes derived from senescent cells can promote cell senescence. The ﬁndings expand the current knowledge on exosomes-mediated aging and provide a novel understanding of the relationship between SASP and senescence. This study is supported by National Natural Science Foundation of China (No. 81771520 and 31702144).

